# Post-traumatic stress disorder among Syrian refugees in Greece

**DOI:** 10.3389/fpsyt.2022.911642

**Published:** 2022-10-13

**Authors:** Dimitrios Theofanidis, Savvato Karavasileiadou, Wafa Hamad Almegewly

**Affiliations:** ^1^Nursing Department, International Hellenic University, Thessaloniki, Greece; ^2^Department of Community Health Nursing, College of Nursing, Princess Nourah bint Abdulrahman University, Riyadh, Saudi Arabia

**Keywords:** post-traumatic stress, refugees, diagnostic and statistical manual of mental disorders, refugee camps, Syria, Greece

## Abstract

**Background:**

Post-Traumatic Stress Disorder (PTSD) is a psychiatric entity developed by those who have been through a traumatic experience. The civil wars in Syria and neighboring countries during the past few years might trigger such experiences, and the same could be argued for the difficult journey from the actual war zones to Europe.

**Purpose:**

To determine the level of PTSD among Arabic-speaking refugees in a Greek refugee camp, who originate primarily from Syria.

**Methods:**

This study involves (*N* = 73) Syrian refugees, all located in Greek camp. Data were gathered using the civilian version of PTSD CheckList (PCL-C). The Arabic version of the PCL-C was used. Individual scores were evaluated *via* use of DSM-IV criteria.

**Results:**

PTSD was found in 58 participants, afflicting both genders (72% of men and 45% of women). In addition, the severity score was recorded to be significantly elevated for the majority of the participants. Finally, the most vulnerable age group was the same for both genders, featuring those between the ages of 35 and 44 years.

**Conclusions:**

The findings of this study demonstrated that the traumatic experiences, suffered by these refugees either before and/or on their journey to Greece, had a severe mental impact. It is imperative that all refugees suffering from this disorder be diagnosed in time and receive appropriate support.

## Introduction

Migration is an umbrella term which is not defined under international law. It reflects an understanding of someone who moves from his/her usual place of residence, whether within a country or across an international border, temporarily or permanently, and for a variety of reasons. Therefore, the term “migration” includes several well-defined legal categories, i.e., ranging from migrant workers to persons whose particular types of movements are legally-defined, such as smuggled migrants (1).

The UNHCR reports that despite the current conflict in Ukraine and other hotzones round the globe, Syria remains the world's largest refugee crisis with 7 million Syrians having fleed their country since 2011 with an additional 6.9 million being internally displaced. The vast majority have found refuge in neighboring countries, primarily in Turkey, Lebanon and Jordan, and Greece with Germany and the Scandinavian countries being the main final destination for further settlement (2).

The consequences of migration for developed countries are complex and diverse, including a wide spectrum of efforts to prepare and adapt to the newcomers to extreme reactions of xenophobia. The health consequences of migration are also diverse, including a variety of minor to very serious health problems. Thus, refugees and migrants often suffer from accidental injuries, hypothermia, burns, gastrointestinal illnesses, cardiovascular events, complicated pregnancy-, and delivery- related complications with the additional burden of psyshological trauma and related conditions (3).

Baird et al. (4) in their study with Syrian war refugee children, have found through a digital analysis of features in children's drawings, great issues of concern regarding their mental health. In this context, Dehnel et al. (5) found a statistically significant positive correlation between trauma and depression, amongst a population of Syrian refugees residing in Jordan with low levels of resilience which may predict further poor mental health outcomes in the future.

In Greece, there is a substantial Syrian refugee population temporarily residing in camps, hoping to be able to move to Northern Europe. To date, there is no published research on the stress levels of Syrian refugees in Greece, thus the need to explore their mental health status.

According to the American Psychiatric Society (6), Post-Traumatic Stress Disorder Syndrome (PTSD) “is a psychiatric disorder that can happen to people who have experienced or witnessed a traumatic event such as a natural disaster, a serious accident, a terrorist attack, war or battle, rape or other attack on that person”. Undoubtedly, war, in itself is commonly associated with ultimate traumatic experiences such as death and destruction, but further psychological trauma may occur due to witnessing of extreme violence, terrorist attacks, kidnappings, torture, separation from family and forced migration whereby the individual has to leave in search of survival (7).

Thus, PTSD can be a dangerous and debilitating mental disorder both for the individual and for society itself. In recent years, the effects of its symptoms have been further investigated and studies in the general population have uncovered its serious impact on the functioning of society in general. According to several studies, there is a plethora of events and situations that can significantly affect a person's mental health. Many times this influence can be controlled by the person himself, without any intervention, through various defensive mechanisms, while sometimes the traumatic events *per se*, can lead directly and swiftly to the formation of PTSD (8, 9).

The International Organization for Migrants defines migration as a movement of a person or group of people either through international borders or within a state, regardless of its size, composition (how many men, women, children are involved, which age group and nationality) and the causes that provoked it (10). The current mass migration phenomenon toward Europe due to the turbulence of the Arab world in recent years and the Syrian civil war in particular, calls for more indepth knowledge regarding the migrants' mental suffering and prevalence of PTSD.

The United Nations High Commission for Refugees (2015) calls for the protection of >5 million Syrian refugees fleeing their country since the beginning of the civil war in 2011. Still, hundreds of refugees reach the European Union (EU) daily and the violence, trauma, and destruction of communities, which many have experienced, have built an urgent need for increased psychosocial and mental health resources within refugee camps (11).

Culturally sensitive treatments targeted for Syrian refugees are recommended for addressing the growing rates of PTSD and depression among residents in camps. Research on promoting resilience for Syrian refugees also recommends advocating for the detection of mental illnesses at early stages, as well as increased efforts in community building and psychosocial support centers. In addition, representatives of international aid organizations are advised to prioritize the development of psychosocial programs for vulnerable groups such as young girls and women within in the camps (12).

PTSD can occur in both adults and children and in general, during a lifetime, about 70% of the general population claims to have experienced a traumatic event and up to 20% of these develop PTSD (13–15). Research has shown that women are slightly more prone to develop it than men. It is of great importance to have a direct diagnosis in children, as stress often affects concentration, memory, attention and decision-making which, if unrecognized, can lead to a highly problematic life in adulthood.

Many studies indicate that most civilian adults and children in war-affected zones, including those in the Middle East, experience at least one traumatic event as a result of war and political conflict (16–18). In particular for Syria, it has been estimated that 3–30% of refugees experience clinical depression with 50–57% experiencing PTSD. Studies with Syrian refugees in camps in Lebanon showed a PTSD prevalence of 35.4% and current depression of 43.9% (19). Similarly, Syrian refugees in Turkey present with PTSD up to 33.5% (20). In these lines, a study by Mahmood et al. (21) showed that more than half of the residents in an Iraqi refugee camp were suffering from PTSD and depression which constitutes a challenging mental health priority especially among female refugees.

By comparison, Western Europe's prevalence rates regarding PTSD seem to be quite low, ranging from 0.7% (Switzerland) to 3% for the UK (22). Furthermore, the USA general population has a PTSD prevalence of 5–12% (23). In Greece, there is no official data for PTSD prevalence, except for a study concerning children survivors of a ship wreck where 52% were found to have PTSD as opposed to 3% in the general school population (24).

Although various studies have shown that the occurrence of PTSD is most common after exposure to war, a phenomenon characteristic of many populations currently fleeing to Greece and other countries, the receiving country may primarily focus on basic refugee needs such as food and shelter, often overlooking complex psychological states (25, 26). The low quality of life that these mentally challenged people may carry increases the use of various health and social services, resulting in rising costs for these societies.

The Syrian civil war is an ongoing multi-sided conflict involving not only domestic national but international forces as well. Since its outbreak in 2011, it has claimed the lives of more than 350,000 civilians while 5.5 million Syrians have left their country. Moreover, a considerable number of refugees have reached the EU, mainly *via* Greek islands. Thus, Greece has been hosting a substantial number of Syrian refugees (27).

Although mental disorders are quite widespread among refugees, most of them are not routinely screened for psychopathology and mental illness. Thus, there is a further burden for their daily living and their social relationships, despite the fact of now residing at a new and safe location (23). PTSD is the most severe psychopathological condition for people who fled a war zone, i.e., such as the Syrian civil war (28).

Thus, many refugees entering Greece, often on makeshift boats from Syrian conflict zones, are at risk of having had or developing PTSD due to their experiences of war and the trauma of the migration process itself. This study, therefore, seeks to find the extent and severity of this mental health condition in Syrian refugees in Greece (where support services are already stretched due to financial restraints).

The PCL-C is the official diagnostic tool for PTSD and no battery of further testing is needed, particularly with highly vulnerable population as citizens fleeing war zones. PTSD isn't diagnosed until at least 1 month has passed since the traumatic event happened and if symptoms of PTSD are present, the health care professional may begin with a readily available, easy to use diagnostic tool, before performing a complete medical history and physical exam.

## Materials and methods

This is a cross-sectional observational survey was conducted at a refugee camp in northern Greece with a population of ~2,000 refugees, of whom 600 were from Syria in the autumn of 2020. A total of 120 refugees from Syria volunteered to participate from this particular sub-population. Therefore, by approaching 120 and finally including 73 people we initially aimed for 20% and finally reached 12.2% of the target population which is a satisfactory sample for a study of this magnitude. Also, our sample included only those migrants in the camp that originated from a war zone.

The prospective sample members were approached onsite to partake to the study on one particular day *via* a face to face invitation. Ninety one men and women after verbal consent were recruited. Five men and three women refused to continue with the study after reading the first few questions. Of the remaining 83 participants, a further 10 failed to complete the questionnaire, therefore only 73 (53 men, 20 women) managed to complete the research tool in full, and only these were used for statistical analysis.

Data were collected *via* the use of the Investigation of PTSD tool, specially designed for citizens (29).

In order to participate, migrants were required to meet specific inclusion criteria as follows: being over 18 years of age, ability to read and understand Arabic, being a recent refugee from Syria due to conflict and war and not having partaken in a PTSD related study during the previous 3 months. It is worth noting that permission to conduct the study was granted *via* all relevant stakeholders, i.e., the host country's officials, and the authorities of this specific refugee center. Participants' anonymity and confidentiality were secured as no names or other form of identification was sought. An equally important aspect of the research process was gaining the consent of the refugees themselves. Still for practical and cultural reasons participants were not asked formally to sign a consent form as this may have been perceived negatively, e.g., signing of a mental health form etc. Instead, a detailed study information sheet in Arabic was presented to them which they were encouraged to keep. Participants were assured that they could stop the study at any point without any repercussions and that refusal to participate had no repercussions whatsoever.

Interviews took place either at the participant's living quarters, i.e., a tent or in some cases a plastic prefabricated shelter or in a community center within the camp. Interviews were conducted with the help of a translator fluent in Greek and Arabic who encouraged respondents closely to follow the questionnaire and to record their answers.

### Research tools

The PTSD Checklist, i.e., PCL (Post-Traumatic-Stress-Disorder Checklist) and specifically PCL-C (Civilian Version) which is an approved and specially designed version of PCL for citizens and was used to diagnose and measure the severity of PTSD in this sample. The Arabic version of the PCL-C was used (30). The PCL-C is a recognized self-report Likert type rating scale for PTSD comprising of 17 items that correspond to the key symptoms of PTSD with responses as follows: 1 = Not at all, 2 = A little bit, 3 = Moderately, 4 = Quite a bit, and 5 = Extremely, giving a total score range of 17–85.

Another advantage of this tool is that it is designed to be completed by the participants without the need for a trained psychologist or other specialist and is usually completed within 10 min. Over the years, the PCL has shown strong psychometric properties, i.e., internal consistency (Cronbach's alpha) range between 0.94 and 0.97. Test-retest reliability is 0.96 at 2–3 days and 0.88 at 1 week (31, 32). The tool correlates positively with the Mississippi PTSD Scale with convergent validity of between *r* = 0.85 and 0.93. The questionnaire has a 97% agreement with the full version of PTSD Clinical Interview and this shorter version was a key factor in the tool selection process (33). Another added advantage is that this questionnaire does not limit the responses of the participants to one specific traumatic event, thus making it highly suitable for our research sample enabling responses to multiple traumatic events.

The main limitation of using PCL-C, instead of a structured clinical interview such as the Clinician Administered PTSD Scale, which is the gold standard for diagnosing PTSD, according to DSM-IV symptom criteria, is that it provides a presumptive diagnosis and it may overestimate PTSD prevalence. The PCL-C is scored in two ways as follows: A total severity score (by simply adding score items) giving a normative threshold of 44 or 50 for indicating a probable diagnosis of PTSD. More specifically, according to Blanchard et al. (34), a cutoff score of 50 for a diagnosis has demonstrated good sensitivity (0.78–0.82) and specificity (0.83–0.86), while a cutoff score of 44 revealed better sensitivity (0.94), specificity (0.86), and overall diagnostic efficiency (0.90). This cut off value was used for this study's needs.

An alternative and perhaps more sophisticated strategy to simply adding individual score items as above, is to compute the responses in two broad categories, i.e., Non-symptomatic (1–2, i.e., “not at all” to “a little bit”) and symptomatic (3–5, i.e., “moderately” or above) followed by DSM-IV criteria for a diagnosis as shown below and used in this study.

- Symptomatic response to at least 1 “B” item, i.e., “Dissociative symptoms” (Questions 1–5),
- Symptomatic response to at least 3 “C” items, i.e., “Intrusion Symptoms” (Questions 6–12), and
- Symptomatic response to at least 2 “D” items “Avoidance Symptoms” (Questions 13–17).

In all cases, it should be noted that a cut off score of 3 or more on the Likert type scale (i.e., moderate or above) for each item on the PCL-C is counted as symptom-present and hence is considered an appropriate scoring technique.

### Data analysis

Data were summarized by computing absolute and relative frequencies (percentages %) and the corresponding 95% confidence intervals (95% CI). A correction for multiple comparisons was not applied, instead, percentages' comparisons between groups of refugees was accomplished with the Fisher's Exact test at *P* ≤ 0.05. All statistical analyses were performed with the IBM SPSS v.21.0 statistical software.

## Results

Of 120 subjects chosen to participate in this study, 91 consented, but 8 (5 men and 3 women) refused to continue with the study after having read the first question for reasons they would not disclose. Out of the 83 who consented (58 men and 25 women), 10 (5 men and 5 women) did not complete the questionnaire, leaving a final sample of 73 participants (53 men and 20 women). There were more males rather than females in the sample, which is mainly due to cultural and other reasons for this sample, as males are possibly more likely to agree in being interviewed or take part in a research study in general.

DSM-IV criteria for PTSD were used in order to answer the fundamental question of this study, i.e., what is the incidence of PTSD in this particular sample. Out of 73 participants, 47 (64.4%, 95% CI: 53.4–75.4%) were diagnosed with PTSD. In terms of gender, 38 men (71.7%, 95% CI: 59.6–83.8%) and 9 women (45.0%, 95% CI: 23.2–66.8%) were diagnosed with PTSD. Fisher's Exact Test showed statistical significance difference between the two genders (*P* = 0.024).

PTSD occurrence according to age groups for both men and women can been seen in Table 1. The data shows that for the 53 men in the sample scores indicating PTSD was high (≥60%) for those aged 18–44 years. Women also followed a similar pattern. However, there is a trend showing that older refugees, i.e., >45 years, showed greater resilience to PTSD. Yet, the small numbers in each age group mean that assertion of this finding is weak. The A series of Fisher's Exact Tests showed that there were no statistically significant differences between the two genders per age group (Table 1) in relation to PTSD occurrence (*P* > 0.10).

**Table 1:** PTSD occurrence relative to gender and age groups.

	***n***	**Diagnosed with PTSD**	**Within Cohort diagnosed with PTSD**	**Fisher's Exact test for gender differences per age group**
**Men**	53	38 (71.7%)	
18–24	14	12 (85.7%)	31.6%	*P* = 0.117
24–34	13	8 (61.5%)	21.1%	*P* = 0.268
35–44	12	11 (91.6%)	28.9%	*P* = 0.176
45+	14	7 (50.0%)	18.4%	*P* = 0.182
**Women**	20	9 (45.0%)		
18–24	6	3 (50.0%)	33.3%	
24–34	7	3 (42.8%)	33.3%	
35–44	5	3 (60.0%)	33.3%	
45+	2	0 (0.0%)	0.0%	
Total	73	47 (64.4%)		

Additionally, the PTSD average score for all participants diagnosed with PTSD was 57 (out of 85 maximum). Thirty-three participants out of 47 (70.2%, 95% CI: 57.1–83.3%) showed relatively high severity of PTSD symptoms (scored between 44 and 57). Eleven men (28.9 95% CI: 14.5–43.3%) and 3 women (33.3%, 95% CI: 2.5–64.1%) had low severity PTSD symptoms.

## Discussion

Approximately 5.6% of a general population will develop PTSD in their lifetime under ordinary social circumstances, i.e., no war, major conflict or other major mass event (35). Our study on Syrian refugees showed a 12-fold greater prevalence in a population coming out of an armed conflict zone. PTSD rates vary widely for refugee populations, with prevalence rates between 4–86% and 5–31% for depression (36). In comparison, a study on Bosnian refugees showed great psychological distress amongst 45% of the study participants meeting criteria for depression, PTSD (37).

Our study was conducted in a makeshift refugee camp in Northern Greece and therefore, the place of data collection should be taken into account for refugee research indicates that living in institutional camps is more disruptive than temporary accommodation with family, friends, or private living. A meta-analysis of 14 different studies has demonstrated that although the general stress of war impacts everyone, displaced persons are significantly more disturbed than non-displaced controls even when the controls had experienced considerable war stress (38).

The large number of refugees arriving in Greece makes recognition of PTSD a priority for health care professionals. Our findings confirm the importance of taking steps to eleviate this distressful condition. The camp nurses alongside the rest of the health care team are therefore important in this area because, with early recognition, stress factors can be identified, and solutions proposed and acted upon. Thereby, the stress experienced and mental hardship could be eased for these vulnerable populations. Thus, awareness and readiness help the healthcare professional in contributing to an insightful approach to the anticipated post-traumatic stress disorder prevalence in refugee camps.

With regard to gender differences in our sample, it was clear that more men than women were prepared to partake in this study. This could be due to the male-dominated nature of this group. However, women account for only 17% of the refugee population coming to Greece so one would expect a smaller number to reflect in our sample.

In our sample, 64.4% presented with PTSD while in another recent survey of Iraqi Yazidis refugees displaced in Turkey, the incidence rate was 42.9% (39). Yet, one should note that exposure to the trecherous passage from Turkey to Greece (as with our sample of refugees) adds a further layer of anxiety and stress (40, 41).

In this sample, men and women with PTSD were 71.7 and 45.0%, respectively. This finding is in contrast with other research in this field which suggests that males are more prone to developing PTSD. However, it should be recognized that women in our sample may have been less exposed to traumatic events and that men between 35 and 44 (who had the highest incidence rate) may have grieved the loss of a previously high quality of life. Nevertheless, both sexes suffered high levels of PTSD.

A case can also be made about the importance of age distribution in the sample of PTSD sufferers. Men of 18–24 years had a high incidence of PTSD compared to those above this age, despite men aged those 35–44 ranking the highest incidence. This finding indicates that younger refugees may be slightly more vulnerable to traumatic events and runs counter to a similar study which showed that higher PTSD rates were observed at about 40 years for men and at 50 years for women (42). Yet, our study also showed that women over 45 years are more likely to show some resilience to PTSD.

Unfortunately, our data did not include levels of stress prior to the actual traumatic event (i.e., the Syrian civil war) because it has been shown that older adults with a history of psychiatric distress or chronic health problems, who are internally displaced, are more likely to endorse significant levels of psychiatric distress (43). The demographical data collected was on the sample's age and gender. As per the study's aim, we wanted to explore the extent and severity of PTSD in Syrian refugees who came from war zones and not to associate this with, e.g., their educational or economic background, as these people did escape from highly conflict situations and once in the boat to Greece or beyond, their status was changed into a refugee. Therefore, our results should be considered under this specific limitation.

Traumatic experiences are often under observed especially within hosting camps where the priority seems to be to cater for people's daily basic needs. Yet, as this study's results have shown, PTSD is an increasingly frequent occurrence in samples coming from conflict zones (44). Under this light, caring for basic life needs is insufficient in itself as these individuals are at additional risk of becoming totally dysfunctional if their disorder is not recognized and dealt with accordingly. Therefore, it is suggested that regular screening for PTSD and other related conditions, to be a routine task especially when admitted to a camp and at a later time at follow up. Furthermore, health care professionals need to be trained in recognizing early signs of PTSD both at an individual and social context. As symptoms caused by trauma and their duration determine the development and appearance of PTSD, nurses working in camps during their daily interaction with migrants and refugees need to be vigilant on spotting alterations in cognition, mood, arousal and reactivity levels. Moreover, camp staff needs to be reminded that they can help to control the symptoms of PTSD with medication which allows many people to participate more effectively in psychotherapy. Drugs like antidepressants (selective serotonin re-uptake inhibitors and serotonin-norepinephrine re-uptake inhibitors) are commonly used to treat the core symptoms of PTSD especially when used in combination with psychotherapy or other treatments.

## Conclusions

This study confirms that there is a serious problem of post-traumatic stress disorder among refugees in a camp in Greece. The finding that almost two thirds of those diagnosed with PTSD had high scores (as shown in the PTSD tool) highlights how much war, the treacherous journey to Europe and the lack of stability combined with anxiety for the future affects the mental health of this Northern Greek sample of refugees.

The findings of this study on Syrian refugees showed a 12-fold greater prevalence of PTSD (as compared to a standard population) in a group which came out of an armed conflict zone *via* a highly uncertain sea transpass, ending up in an even more uncertain camp in Greece. Therefore, the needs of these people are multiple, with special psychological and social support required.

## Study limitations

Certain limitations apply to this study, starting from the relatively small sample size which although it might be adequate for a single camp, the generalizability of our results to the whole Syrian refugee population in Greece is not possible. Also, there was a dropout rate concern which would need to be considered should this study be followed up, i.e., to devise strategies in preventing this. Therefore, further research is needed in order to clarify and explain further on the absolute prevalence of PTSD amongst this vulnerable group of people.

## Data availability statement

The raw data supporting the conclusions of this article will be made available by the authors, without undue reservation.

## Ethics statement

The studies involving human participants were reviewed and approved by International Hellenic University. The patients/participants provided their written informed consent to participate in this study.

## Author contributions

SK and DT designed the study and analyzed the data. DT did the literature review and collected the data. WA prepared the manuscript. All authors contributed and approved the final manuscript.

## Funding

This work was supported by Princess Nourah bint Abdulrahman University Researchers Supporting Project Number (PNURSP2022R312), Princess Nourah bint Abdulrahman University, Riyadh, Saudi Arabia.

## Conflict of interest

The authors declare that the research was conducted in the absence of any commercial or financial relationships that could be construed as a potential conflict of interest.

## Publisher's note

All claims expressed in this article are solely those of the authors and do not necessarily represent those of their affiliated organizations, or those of the publisher, the editors and the reviewers. Any product that may be evaluated in this article, or claim that may be made by its manufacturer, is not guaranteed or endorsed by the publisher.

## Abbreviations

DSM-IV: Disease Statistical Manual-IV
EU: European Union
PCL-C: Post-Traumatic-Stress-Disorder Checklist
PTSD: Post-Traumatic Anxiety Disorder Syndrome.
